# Integrated GPS-Enabled Physical Activity and Dietary Interventions Versus Physical Activity Alone for Obesity Control: A Systematic Review and Meta-Analysis

**DOI:** 10.3390/nu17111886

**Published:** 2025-05-30

**Authors:** Yu Fan, Sichen Zhang, Xiaomin Sun, Zhaozhang Sun, Wen Peng, Lin Shi, Bo Gou, Youfa Wang

**Affiliations:** 1 Global Health Institute, School of Public Health, Xi’an Jiaotong University Health Science Center, Xi’an 710061, China; fiona.yiyi579@gmail.com (Y.F.); 17355324065@163.com (S.Z.); gzhtxiaomin@163.com (X.S.); z.sun.4@bham.ac.uk (Z.S.); 2 Department of Applied Health Sciences, School of Health Sciences, College of Medicine and Health, University of Birmingham, Birmingham B15 2TT, UK; 3 Centre for National Training and Research Excellence in Understanding Behaviour (CENTRE-UB), University of Birmingham, Birmingham B15 2TT, UK; 4 Department of Public Health, Qinghai University Medical College, No. 16 Kunlun Rd, Xining 810008, China; wen.peng2014@fox-mail.com; 5 School of Food Engineering and Dietary Science, Shaanxi Normal University, Xi’an 710119, China; linshi198808@snnu.edu.cn; 6 Key Laboratory of Sports Technology Analysis and Skill Assessment General Administration of Sport, Xi’an Physical Education University, Xi’an 710068, China; 103014@tea.xaipe.edu.cn

**Keywords:** GPS-enabled interventions, physical activity, dietary, obesity control, digital health, meta-analysis, dietary intervention, wearable technology

## Abstract

**Background:** The escalating prevalence of obesity underscores the urgent need for effective and scalable interventions. Global Positioning System (GPS)-enabled technologies have emerged as promising strategies to promote physical activity (PA) and address obesity. However, the comparative effectiveness of GPS-enabled PA interventions integrated with dietary components versus PA interventions alone remained unclear. This study aimed to systematically evaluate and compare the effectiveness of GPS-enabled PA interventions, with or without dietary strategies, in improving obesity-related outcomes among adults. **Methods**: A systematic review and meta-analysis was conducted in accordance with PRISMA guidelines. Randomized controlled trials (RCTs) published between January 2000 and April 2025 were retrieved from five databases. Eligible studies included GPS-enabled PA interventions targeting adults (≥18 years old), and reported at least one primary obesity-related outcome. Meta-analyses were performed using random- or fixed-effects models, depending on heterogeneity levels, and subgroup analyses explored effect modifiers. **Results**: Nine studies (involving 1363 participants, 424 males and 939 females, aged from 34.5–64.8) were included. GPS-enabled PA interventions significantly reduced body weight (Hedges’ g = −0.241, 95% CI: −0.356 to −0.127, *I*^2^ = 6.5%, Q = 7.49, *p* = 0.380) and body fat percentage (BFP) (Hedges’ g = −0.412, 95% CI: −0.804 to −0.020, *I*^2^ = 76.0%, Q = 16.66, *p* = 0.002). Subgroup analyses revealed that interventions involving PA alone produced a moderate effect on weight reduction (Hedges’ g = −0.328; 95% CI: −0.616 to −0.039), whereas those combining PA with dietary strategies showed a slightly smaller yet significant effect (Hedges’ g = −0.208; 95% CI: −0.372 to −0.044). Short-term interventions (≤3 months) demonstrated greater effects on weight reduction. Sensitivity and bias assessments supported the robustness of short-term outcomes. **Conclusions**: GPS-enabled PA interventions were effective for promoting short-term reductions in body weight and BFP. Notably, the addition of dietary components did not consistently provide greater benefits compared to PA interventions alone. These findings highlight the utility of geospatial technology in enhancing behavioral interventions and support the development of scalable digital health strategies aligned with public health priorities such as “Healthy China 2030”.

## 1. Introduction

The global rise in overweight people and obesity has become a critical public health concern, driving the growing burden of chronic diseases such as diabetes, cardiovascular disorders, and metabolic syndromes [1]. The increasing prevalence highlights the urgent need for effective and sustainable weight management strategies. Among these, physical activity (PA) has consistently been identified as a cornerstone intervention, particularly when combined with dietary modifications and behavioral counseling [2]. However, maintaining long-term adherence to such lifestyle changes remains challenging, often hindered by motivational, environmental, and structural barriers [3].

In response to these challenges, there has been increasing interest in leveraging digital technologies to facilitate engagement in PA. One such promising innovation is the application of Global Positioning System (GPS) technology within PA interventions [4]. GPS enables high-resolution tracking of individuals’ spatial and temporal movement patterns in free-living conditions, providing objective insights into mobility behaviors [5]. When integrated with smartphone applications for dietary tracking and self-monitoring, GPS-based systems offer dynamic features such as real-time feedback, adaptive goal setting, and social support—components known to improve behavioral adherence and long-term engagement [5,6]. While GPS-based PA strategies provide promising avenues for promoting physical activity, nutritional intake remains another critical determinant of energy balance and weight management, particularly when integrated with digital interventions.

In addition to physical activity, dietary intake plays a fundamental role in regulating energy balance and body composition [7]. Obesity typically results from a sustained positive energy balance, where energy intake exceeds expenditure [8]. Nutritional interventions—such as caloric restriction, structured diet plans, or app-assisted dietary tracking—have been shown to enhance weight control when combined with physical activity [9]. Evidence from large-scale reviews consistently demonstrates that multicomponent programs integrating both diet and PA achieve superior outcomes. For example, Johns et al. (2014) [10] found that combined behavioral interventions were significantly more effective in reducing body weight than diet-only or PA-only strategies. Similarly, a Cochrane systematic review confirmed that such multicomponent interventions outperform single-modality approaches in both efficacy and sustainability [9,11].

However, the magnitude of these additive effects may be influenced by factors such as adherence to dietary protocols, digital literacy, feedback frequency, and the complexity of self-monitoring tools [12]. In digital health interventions—especially those using GPS-enabled platforms—these variables may moderate behavioral outcomes [13]. Therefore, rather than treating dietary components as a homogeneous construct, it is methodologically important to examine them as distinct modifiers [14]. This rationale underpins our decision to perform subgroup analyses comparing GPS-enabled PA-alone interventions with those combining PA and dietary strategies.

Emerging evidence has demonstrated the value of GPS-enabled tools for contextualizing PA within the environmental landscape. For instance, Marquet et al. [15] reported that greater walkability and greenness in GPS-defined activity spaces were positively associated with higher levels of moderate-to-vigorous PA and step counts. Similarly, Liu and colleagues [16] developed a device-agnostic GPS–accelerometer integration algorithm that accurately distinguished indoor versus outdoor time, achieving over 89% accuracy. These findings highlight how GPS can be utilized to capture context-sensitive activity patterns, supporting scalable monitoring. However, evidence from GPS-based interventions targeting individual-level weight loss remains mixed, and few studies have systematically examined how variations in intervention content (e.g., PA only vs. PA combined with dietary) influence weight-related outcomes. Although dietary behaviors and PA are theoretically complementary in weight control, scientific evidence on their combined effects remains somewhat heterogeneous. A systematic review by Johns et al. [10] showed that behavioral interventions combining diet and PA resulted in greater weight loss than single-component interventions. Similarly, a Cochrane meta-analysis [11] confirmed that multicomponent behavioral programs generally outperform standalone PA or diet strategies. Yet the magnitude of additive effects appears to vary depending on intervention duration, intensity, participant characteristics, and adherence levels. Whether these additive benefits are preserved in GPS-enabled, technology-mediated interventions remains unclear [17].

To address this gap, we conducted a systematic review and meta-analysis of GPS-enabled PA interventions targeting on weight management outcomes (e.g., body weight, body mass index [BMI], waist circumference, waist–hip ratio [WHR]). We further compared the effects of interventions that combined PA with dietary components to those that used PA alone. The findings aim to support the development of scalable interventions that employ emerging technologies to increase PA and improve weight management outcomes.

## 2. Materials and Methods

This study was conducted in accordance with the Preferred Reporting Items for Systematic Reviews and Meta-Analyses (PRISMA) guidelines and the Cochrane Collaboration handbook for Systematic Reviews of Interventions [18]. The study protocol was registered on the International Prospective Register of Systematic Reviews (PROSPERO, CRD 42024545777).

### 2.1. Search Strategy

A comprehensive systematic search was conducted to identify randomized controlled trials (RCTs) evaluating the effects of GPS-enabled behavioral interventions on physical activity and weight-related outcomes among adults aged 18 years and older. A comprehensive search was performed across five electronic databases: PubMed, Embase, Scopus, Web of Science, and Medline for studies published from 1 January 2000 to 1 April 2025.

Search strategies combined Medical Subject Headings (MeSH) and free-text terms using Boolean operators (AND, OR) across four key conceptual domains: (1) GPS and digital technologies (“Geographic Information Systems” OR “Global Positioning Systems” OR “Digital Health” OR “Mobile Health” OR “Wearable Devices” OR “Health Wearables” OR “Consumer Wearables”); (2) physical activity behaviors (“Exercise” OR “Physical Activity” OR “Lifestyle” OR “Aerobic Exercise” OR “Exercise Training” OR “Step Counts” OR “Intensity” OR “Duration”); (3) study populations (“Adult” OR “Individuals” OR “Participants” OR “Population”); and (4) obesity-related outcomes and study design (“Overweight” OR “Obesity” OR “Obese” OR “Body Weight” OR “Body Mass Index” OR “Body Fat” OR “Waist Circumference” OR “Waist-hip Ratio”) AND (“Randomized Controlled Trial” OR “Intervention Study” OR “Controlled Clinical Trial”). Additionally, manual searches of the reference lists of included articles were conducted to capture any additional relevant studies not identified through database searches. Grey literature (e.g., dissertations, unpublished reports, conference abstracts) was not systematically searched.

### 2.2. Study Selection

The published English full-text studies were included if they met all the following criteria: (1) were GPS-enabled RCTs and reported sufficient data for effect size calculation; (2) subjects were ≥18 years old; (3) were required to explicitly incorporated behavioral change functionalities (e.g., geofencing, real-time activity tracking, or location-triggered prompts); and (4) reported at least one primary obesity-related outcome (body weight, BMI, BFP, waist circumference, or WHR).

Exclusion criteria included the following: (1) focused solely on wearable devices for non-behavioral tracking (e.g., dietary tracking without a focus on changing PA behaviors); (2) were protocol papers without completed outcome data; (3) primarily compared different GPS technologies or used GPS as part of a broader multi-component intervention without isolating the GPS-based PA effect; and (4) involved a single time-point assessment (baseline or follow-up only), or studies reporting only secondary analyses.

### 2.3. Data Extraction and Study Quality Assessment

#### 2.3.1. Data Extraction

Two reviewers independently extracted data from each included study using a standardized extraction form. Extracted information compassed study characteristics (authors, year of publication, country, population, sample size, participant demographics including mean age, BMI, and gender), intervention features (type and model of GPS incorporated devices/techniques), and behavioral components of the intervention. The second reviewer independently verified all extracted data for completeness and accuracy. Any discrepancies were resolved through consensus or consultation with a third reviewer.

#### 2.3.2. Study Quality Assessment

The risk of bias for all included studies was assessed independently by two reviewers using the Cochrane Collaboration’s Risk of Bias Tool (RoB 1.0), as recommended in the Cochrane Handbook [19] for Systematic Reviews of Interventions. The following seven domains were evaluated: (1) random sequence generation; (2) allocation concealment; (3) blinding of participants and personnel; (4) blinding of outcome assessment; (5) incomplete outcome data; (6) selective reporting; and (7) other potential sources of bias.

Each domain was judged as “low risk” “high risk” or “unclear risk” based on explicit criteria provided in the Cochrane Handbook. Discrepancies between reviewers were resolved by discussion or arbitration by a third reviewer if necessary. Consistent with established practice, risk of bias judgments were not used to exclude studies from the meta-analysis, but were considered in the interpretation of findings, particularly during sensitivity and subgroup analyses [20].

### 2.4. Data Analysis

All meta-analysis and subgroup analyses were performed using Stata version 17.0 (Stata Corp LLC, College Station, TX, USA). Statistical significance was set at a two-tailed *p*-value of <0.05. The effect size was calculated using Hedges’ g with corresponding 95% confidence interval [21]. Statistical heterogeneity was assessed using the *I*^2^ statistics (categorized as ≤25% low, 25–50% moderate, 50–75% substantial, >75% considerable). A fixed-effects model was applied when heterogeneity was (*I*^2^ ≤ 25%), while a random-effects model was used for moderate to considerable heterogeneity (*I*^2^ > 25%) [22].

Subgroup analyses were conducted to evaluate the intervention effects by intervention type, participant gender, and age group. Sensitivity analyses were conducted by excluding one study at a time from the meta-analysis. Publication bias was assessed utilizing visual inspection of funnel plots, along with Egger’s regression test and Begg’s rank correlation test. A two-tailed *p*-value < 0.05 was considered indicative of statistically significant publication bias [23].

## 3. Results

### 3.1. Main Study Characteristics and Findings

**Figure 1:**
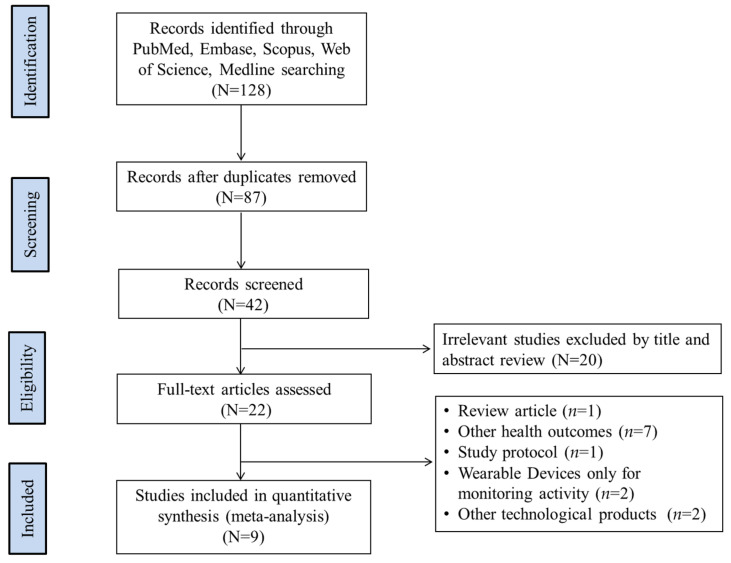
Flowchart of literature search and assessment of articles.

A total of 128 records were initially identified. After screening, 22 full-text articles were assessed for eligibility [24,25,26,27,28,29,30,31,32,33,34,35,36,37,38,39,40,41,42,43,44,45]. Of these, 13 full-text articles were excluded for the following reasons: irrelevant outcomes (*n* = 7), use of wearable devices for monitoring only without intervention (*n* = 2), protocol-only publications (*n* = 1), review articles (*n* = 1), and interventions based on non-GPS technologies such as web-based platforms or video games (*n* = 2). Finally, nine studies met the inclusion criteria and were included in the meta-analysis [24,25,26,27,28,29,30,31,32] (Figure 1).

Table 1 summarizes the key characteristics of the included studies. All studies were published between 2015 and 2024, and were conducted across seven countries: Spain (*n* = 2), South Korea (*n* = 1), Netherlands (*n* = 1), Sweden (*n* = 1), Belgium and Ireland joint study (*n* = 1), Canada (*n* = 1), and the United States (*n* = 2). Sample sizes varied substantially, ranging from 20 to 440 participants, with a median of 110. The mean age of participants ranged from 34.5 to 64.8 years, and the proportion of female participants ranged from 18.3% to 100%.

All interventions incorporated PA components, with five studies additionally incorporating structured dietary interventions [24,25,27,28,32]. Intervention durations ranged from 3 to 12 months. The intervention group employed wearable technologies or mobile applications for behavior tracking, such as pedometer apps, smart bands, accelerometers, or activity monitors. The control group typically received standard care, including general lifestyle counseling or education, without access to digital feedback or structured PA support.

All nine studies assessed obesity-related outcomes, with at least one of the following reported: body weight (*n* = 8), BMI (*n* = 5), BFP (*n* = 5), fat mass (*n* = 3), or waist–hip ratio (WHR) (*n* = 3) (Table A1). The majority of interventions led to favorable changes in weight-related outcomes. Specifically, five studies reported significant reductions in fat mass or BFP, while three reported reductions in BMI. For example, Hernández-Reyes et al. (2020) [24] observed a significant reduction in fat mass without concurrent changes in BMI or body weight. Similarly, Lugones-Sanchez et al. (2020) [25] and Choi et al. (2023) [26] reported meaningful improvements in both BFP and fat mass with the use of smart bands or AI-driven mHealth systems. In contrast, studies with lower intervention intensity or without dietary components tended to show limited or non-significant improvements in BMI and body weight.

Notably, two studies (Claes et al., 2020; Uijl et al., 2023) [27,29] emphasized the maintenance of cardiometabolic parameters rather than absolute weight loss, highlighting the role of PA interventions in preserving metabolic stability. Overall, the intervention group demonstrated consistent benefits for obesity control, with more variable effects on different obesity-related outcomes.

### 3.2. Meta-Analysis: GPS-Enabled Effects on Obesity-Related Outcomes

#### 3.2.1. Overall

The meta-analysis demonstrates that GPS-enabled PA interventions incorporating PA, with or without additional dietary components, were associated with modest but potentially meaningful improvements in obesity-related outcomes.

**Figure 2:**
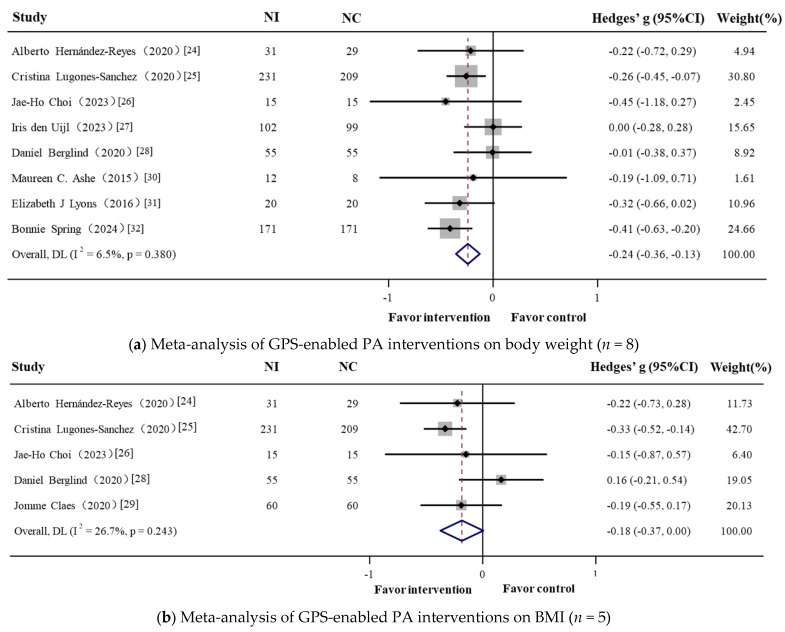

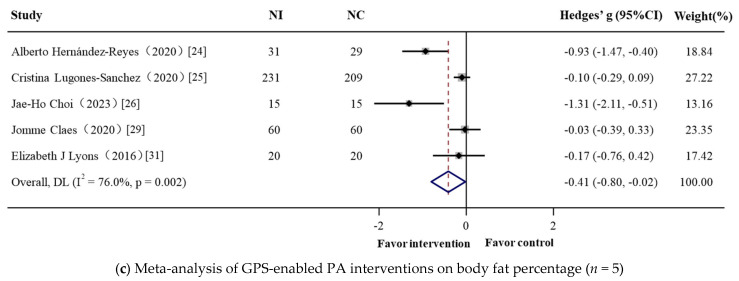
Forest plot of the effectiveness of physical activity in GPS-enabled strategies on body weight ((**a**), *n* = 8), BMI ((**b**), *n* = 5), and body fat percentage ((**c**), *n* = 5). NI: intervention group sample; NC: control group sample. Negative Hedges’ g values indicate a higher score in outcomes in favor of the intervention group.

Compared with control groups, GPS-enabled PA intervention groups had a significant reduction in body weight (Hedges’ g = −0.241, 95% CI = −0.356 to −0.127), with low heterogeneity (*I*^2^ = 6.5%, Q = 7.49, *p* = 0.380). Although there was a trend toward improvement in BMI, the effect did not reach statistical significance (Hedges’ g = −0.185, 95% CI = −0.375 to 0.005), with moderate heterogeneity (*I*^2^ = 26.7%, Q = 5.46, *p* = 0.243). A significant reduction in BFP was observed (Hedges’ g = −0.412, 95% CI = −0.804 to −0.020), although substantial heterogeneity was noted across studies (*I*^2^ = 76.0%, Q = 16.66, *p* = 0.002) (Figure 2).

#### 3.2.2. Subgroup Analysis

**Figure 3:**
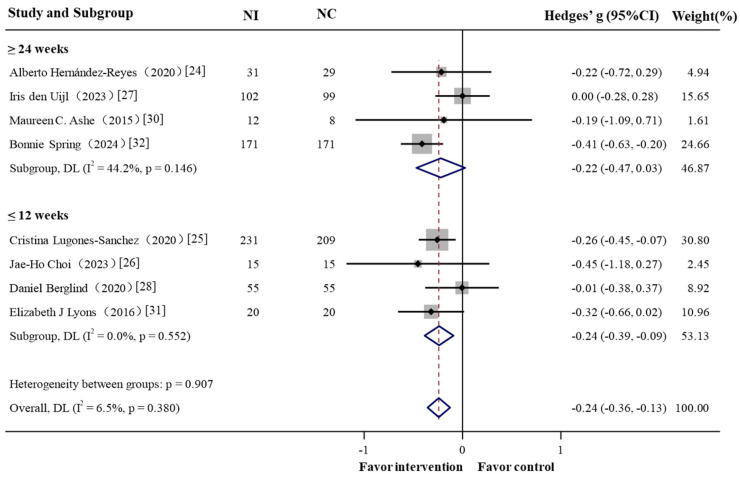
Forest plot of the effectiveness of physical activity in GPS-enabled strategies on body weight change by interventional duration, NI: intervention group sample; NC: control group sample. Negative Hedges’ g values indicate a higher score in outcomes in favor of the intervention group.

**Figure 4:**
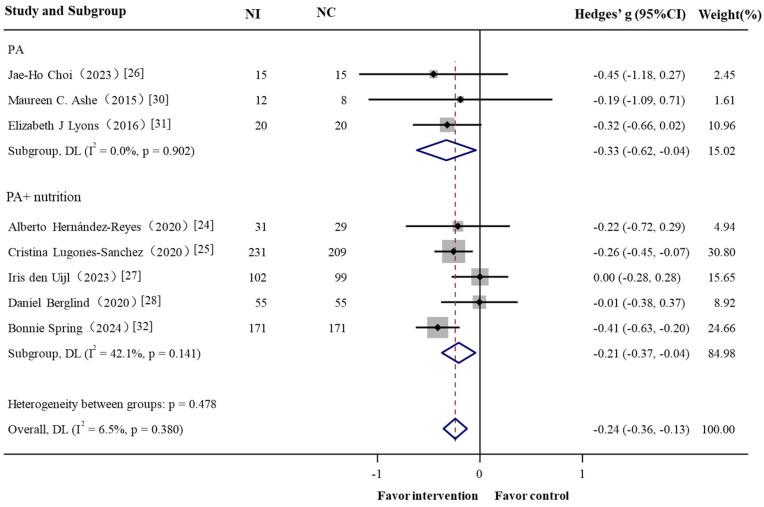
Forest plot of the effectiveness of physical activity in GPS-enabled strategies on body weight change by type of intervention. NI: intervention group sample; NC: control group sample. Negative Hedges’ g values indicate a higher score in outcomes in favor of the intervention group.

In the subgroup analyses, the short-term effect (≤3 months) resulted in greater reduction in body weight (Hedges’ g = −0.239, 95% CI = −0.386 to −0.092) (Figure 3). Interventions involving PA alone produced a Hedges’ g of –0.328 (95% CI = −0.616 to −0.039), which was comparable to the effect observed in interventions combining with dietary strategies (Hedges’ g −0.208, 95% CI = −0.372 to −0.044) (Figure 4).

Stratification by baseline age demonstrates greater reductions in body weight among participants aged ≤60 years (Hedges’ g = −0.221, 95% CI = −0.372 to −0.070). Studies involving only female participants demonstrate comparatively lower weight loss efficacy than those with mixed-gender samples (Hedges’ g = −0.224, 95% CI: −0.381 to −0.067). However, female-only interventions achieved significantly greater improvements in BFP (Hedges’ g = −1.051; 95% CI = −1.495 to −0.606) compared to mixed-gender interventions (Hedges’ g = −0.090; 95% CI = −0.250 to 0.070) (Table A2).

### 3.3. Risk of Bias Assessment

**Figure 5:**
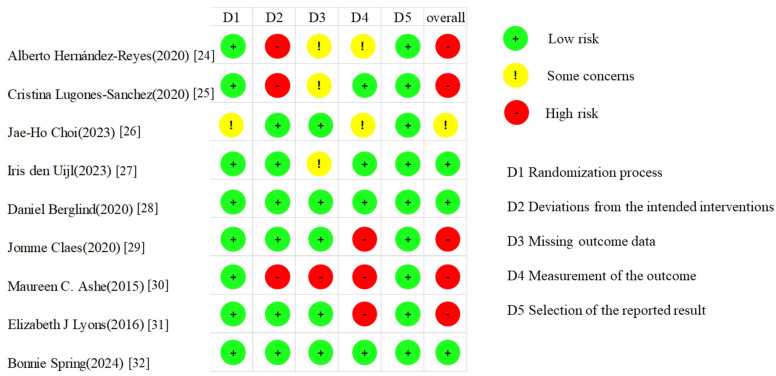
Summary of the risk of bias assessment of the included studies performed by using the Cochrane Collaboration tool.

The overall methodological quality of the included studies was moderate. Four studies demonstrated low or unclear risk of bias in domains such as random sequence generation and selective outcome reporting. However, four studies were judged to have high risk of bias with regard to allocation concealment and blinding of outcome assessment (Figure 5).

### 3.4. Sensitivity Analyses

Sensitivity analyses were conducted to assess the robustness of pooled estimates (Table A3). For body weight, effect sizes remained consistent across all leave-one-out analyses, indicating high result stability. In contrast, the results for BMI change were sensitive to the inclusion of specific studies, particularly those by Cristina (2020) [25] and Jomme (2020) [29], which accounted for most of the observed heterogeneity.

For BFP, sensitivity analyses revealed that studies by Alberto Hernández-Reyes (2020) [24] and Jae-Ho Choi (2023) [26] were primary key contributors to heterogeneity. Excluding these studies reduced *I*^2^ from 75.0% to 66.0% and 66.5%, respectively, suggesting that variability in study design or population characteristics may have contributed to between-study differences

### 3.5. Publication Bias

**Figure A1:**
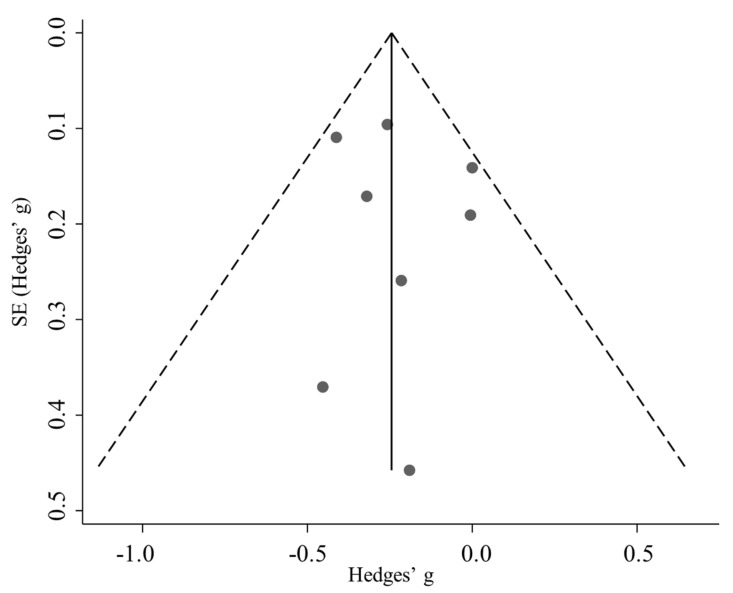
Assessment of publication bias by funnel plot. Each dot represented an individual study. The vertical solid line indicated the overall effect size (Hedges’ g), and the dashed lines represented the 95% confidence limits. Symmetry suggested low risk of publication bias.

There was no indication of publication bias for obesity-related outcomes as indicated by funnel plots (Figure A1). Egger’s and Begg’s tests for body weight (Egger *p* = 0.698, Begg *p* = 0.902), BMI (Egger *p* = 0.201, Begg *p* = 0.462), and BFP (Egger *p* = 0.154, Begg *p* = 0.211) (Table A4) reveal no statistically significant evidence of publication bias.

## 4. Discussion

This meta-analysis provides scientific evidence supporting the effectiveness of GPS-based PA interventions, particularly those integrating PA and dietary strategies, in achieving statistically significant short-term reductions in body weight. The pooled effect size suggests a modest meaningful benefit, consistent with prior reviews highlighting the potential of digital tools incorporating location-awareness functionalities in promoting behavior change [46,47,48]. While multicomponent interventions combining dietary and PA strategies have shown theoretical and empirical advantages [9,11], our findings suggest that GPS-enabled PA-alone interventions yielded comparable or even greater improvements in weight-related outcomes compared to combined PA + diet programs. This counterintuitive result may reflect several contextual and intervention-specific factors observed across the included studies.

Unlike conventional digital interventions, GPS-enabled technologies provide real-time location tracking, geofencing, and feedback grounded in environmental context, facilitating timely, context-specific prompts and behavioral nudges over the past ten years [49]. Their ecological validity and spatial personalization have been shown to significantly improve adherence and promote PA in both experimental and real-world settings [50]. Such systems exhibit high ecological validity by facilitating the seamless integration of physical activity into users’ daily routines—through strategies such as location-triggered notifications and route-specific goal suggestions. This geospatial responsiveness distinguishes GPS-enabled platforms from traditional “smart” devices, which rely predominantly on inertial sensors or self-reported data and lack dynamic adaptation to the user’s physical surroundings [51]. However, there remains a relative paucity of systematic evaluations focusing specifically on the role of GPS-enabled PA interventions in weight management.

First, the comparative analysis of GPS-enabled PA interventions, delivered either alone or in combination with dietary strategies, reveals important insights into their relative effectiveness for weight management. Addressing both sides of the energy balance equation—increasing energy expenditure through PA and reducing energy intake via diet—remains a foundational principle in weight-control interventions [10]. It is widely assumed that integrating dietary modifications with PA should yield superior outcomes, but this meta-analysis suggests otherwise, indicating that GPS-enabled PA interventions alone may achieve comparable, and in some cases greater, improvements in weight-related metrics such as body weight, BMI, and BFP. Dietary components were typically delivered via basic self-report tools or standalone nutrition apps lacking dynamic behavioral feedback, such as real-time progress monitoring, adaptive goal adjustment, or social reinforcement. Studies such as Hernández-Reyes [24] and Choi [26], for instance, employed structured PA interventions supported by feedback-rich systems (e.g., pedometer apps with notifications or AI-enhanced mHealth platforms), while dietary interventions were often limited to scheduled counseling or passive food logging apps, potentially reducing user engagement and adherence. This observation aligns with behavioral science theories suggesting that single-modality interventions may foster better adherence due to reduced cognitive load and lower complexity [13,52].

Moreover, participants enrolled in most GPS-enabled-PA-alone interventions were generally more digitally literate and physically autonomous—typically working-age adults or women without significant comorbidities—making them more receptive to technology-mediated PA strategies. In contrast, several GPS-enabled PA + diet studies (e.g., den Uijl, 2023) [27] targeted populations with cardiometabolic diseases or older adults undergoing rehabilitation, groups inherently more susceptible to cognitive, motivational, or technological challenges when engaging with complex dietary tracking systems. This user-level heterogeneity is consistent with established digital health frameworks, which underscore the importance of tailoring intervention components to users’ cognitive and contextual capacities [14].

In addition, a review of the dietary components within GPS-enabled PA + diet interventions indicates that nutritional strategies were often delivered via low-intensity modalities, including static food logging applications or infrequent, non-personalized counseling sessions [12,26,27]. These approaches typically lacked dynamic features such as individualized goal adjustment, real-time feedback, or contextual prompts. Conversely, PA components were more frequently integrated with interactive features—such as step-count feedback, adaptive targets, or GPS-enabled activity cues—which likely contributed to higher adherence rates. This imbalance in behavioral engagement may have diminished the additive value of dietary strategies. Indeed, limited or absent reporting of dietary adherence was common, and available data indicate modest compliance levels. Prior research in the literature has shown that passive or cognitively demanding dietary monitoring systems are associated with reduced long-term engagement and diminished intervention effectiveness [12]. Collectively, these implementation discrepancies suggest that the delivered “behavioral dose” of dietary intervention was likely subtherapeutic, thereby attenuating the expected synergistic effects in GPS-enabled PA + diet conditions.

Furthermore, GPS-enabled tools, with their real-time tracking, geofencing, and personalized feedback mechanisms, which facilitate seamless integration of physical activity into daily routines and enhance behavioral engagement [53]. In contrast, the addition of dietary components often introduces variability in adherence and effectiveness, due to factors such as dietary literacy, intervention burden, and heterogeneity in dietary protocols [12]. However, few studies including those in our review provided detailed information on participant adherence. This remains a critical gap that future research should address to enhance the reliability and applicability of intervention findings. A significant reduction in BFP was observed, whereas the downward trend in BMI did not reach statistical significance, possibly due to the limited number of studies reporting this outcome. Notably, BFP data were mainly obtained from studies involving female participants, who may respond more readily due to higher baseline fat levels. Moreover, the relatively short duration of interventions may have further constrained the ability to detect meaningful changes [54].

Second, intervention duration emerged as a critical factor moderating intervention effectiveness. A systematic review indicated that short-term, multicomponent interventions (lasting six months or less) were effective in achieving weight loss among adults with obesity or classed as overweight [55]. This pattern likely reflects broader trends observed in digital health interventions, where participant engagement typically declines over time due to motivation decay and diminished novelty effects. Our review extends these insights by demonstrating that the most pronounced weight loss effects occurred within the first three months of GPS-enabled PA interventions (Hedges’ g = −0.286, *p* < 0.001), while outcomes at six months were attenuated and not statistically significant (Hedges’ g = −0.221, *p* = 0.080). This difference may reflect not only the natural decline in participant engagement over time—a phenomenon well-documented in digital health interventions—but also the limited number of long-term studies included in our analysis (*n* = 4 for six months vs. *n* = 4 for three months), reducing the statistical power to detect longer-term effects. Importantly, many of the six-month interventions lacked continuous adaptive support mechanisms (e.g., tailored feedback or social connectivity features), which have been shown to sustain behavioral changes beyond the initial engagement period [14]. These findings reinforce the idea that short-term interventions (particularly within three months) may optimize weight loss outcomes, likely benefiting from higher user retention, novelty effects, and intensified behavioral engagement. Taken together, these findings highlight that the apparent superiority of PA-alone interventions observed in this meta-analysis should not be interpreted as undermining the role of diet. Rather, it reflects the differential implementation quality and contextual integration of PA and dietary components within GPS-enabled digital frameworks. Future GPS-enabled PA interventions should prioritize integrating adaptive reinforcement strategies, social support components, and seamless alignment with broader lifestyle routines to sustain long-term efficacy [56,57].

Third, assessment of publication bias revealed no statistically significant asymmetry based on Egger’s and Begg’s tests, however, the relatively small number of included studies per outcome (*n* < 10) limits the statistical power of these evaluations and necessitates cautious interpretation [58]. Notably, in the subgroup analysis of BFP, heterogeneity among mixed-gender studies was completely eliminated (*I*^2^ = 0.0%) following stratification by sex. This finding suggested that gender composition may moderate intervention effects, potentially masked in aggregated analyses that fail to disaggregate by sex. These findings are consistent with previous evidence suggesting sex-based differences in response to weight loss interventions, potentially due to hormonal profiles, metabolic flexibility, psychosocial determinants, or engagement patterns [58,59]. Nonetheless, caution is warranted, as the small number of female-only studies (*n* = 2) limits the robustness of these conclusions.

Finally, sensitivity analyses revealed that long-term outcomes for body weight and BFP were disproportionately influenced by a small number of studies, including those by Hernández-Reyes (2020) [24] and Choi (2023) [26], both of which employed relatively intensive interventions with structured feedback and personalized activity goals. These studies also tended to involve female-only samples and shorter durations, consistent with our stratified findings showing enhanced intervention effects in these contexts. Future studies should account for these variables in both design and analysis phases. Specifically, stratified reporting by sex, planned subgroup analyses, and transparent disclosure of intervention intensity and engagement strategies will be essential to clarify heterogeneous effects and advance tailored intervention design.

This study comprehensively evaluated GPS-enabled PA interventions for weight management, addressing a critical gap in prior digital health research by integrating real-time spatial tracking with behavior change strategies. Through rigorous synthesis and subgroup analyses, the review not only assessed overall intervention efficacy but also dissected effect modifiers such as intervention type, duration, age, and sex, offering valuable insights for precision-tailored intervention design. The findings underscore the public health relevance and scalability of GPS-enabled PA interventions, particularly in supporting global initiatives like “Healthy China 2030”, which prioritize personalized, technology-driven strategies for health promotion. However, several limitations must be acknowledged. From 2000 to 2025, only nine small-scale RCTs, involving a total of 1363 participants, were eligible for inclusion in our review, highlighting the limited application of GPS-enabled PA interventions for weight management. This scarcity likely reflected several practical barriers rather than a lack of research interest. GPS technologies typically require more advanced hardware, higher energy consumption, and greater costs compared to standard wearable technologies, thereby limiting their scalability in clinical-based trials [53]. Furthermore, continuous location tracking raises privacy and data security concerns, potentially deterring participant enrollment and engagement [60]. The external validity of this review is constrained by substantial heterogeneity across study populations, intervention modalities, and geographic settings. Notably, the majority of included trials predominantly enrolled adults with obesity or classed as overweight, characterized by a mean baseline age of approximately 50.1 years and an average BMI of 30.4 kg/m^2^. This demographic homogeneity may limit the extrapolation of the findings to broader populations, including younger individuals, those with normal weight, or cohorts from different sociocultural and geographic backgrounds. These challenges must be carefully addressed in future research and implementation efforts to fully harness the potential of GPS-enabled strategies for scalable public health impact. Future research should explore the potential of integrating machine learning algorithms to enhance the personalization of GPS-enabled interventions and to dynamically tailor PA prompts in response to real-time behavioral and environmental data. In addition, long-term, large-scale RCTs are needed to validate the sustainability, scalability, and cost-effectiveness of these technologies across diverse populations and settings.

## 5. Conclusions

In conclusion, this study provided a comprehensive evaluation of GPS-enabled PA interventions, marking one of the first systematic efforts to highlight the distinct contributions of geospatial functionalities to weight management outcomes. Our findings suggest that GPS-enabled interventions, particularly in short-term applications, could achieve meaningful reductions in body weight and body fat percentage (BFP). Future research should focus on standardizing intervention protocols, extending durations, and optimizing user-centered designs to maximize the potential of geospatial data for personalized and scalable behavior change. In parallel, policy integration and infrastructure support will be critical to ensure equitable access, protect data privacy, and maximize the public health impact of GPS-enabled technologies, aligning with national health priorities such as the “Healthy China 2030” initiative.

## Figures and Tables

**Table 1:** Characteristics of study populations and intervention approaches of the 9 included studies.

Study	Country	Population	Sample Size (*n*)	Mean Age (years)	BMI (Mean ± SD) (kg/m^2^)	Sex N (%)	Intervention Design
Alberto Hernández-Reyes (2020) [24]	Spain	Adult women with obesity or classed as overweight	60	41.5	31.8 ± 5.3	IG: 31 female (100.0%) CG: 29 female (100.0%)	Approach of intervention: PA + diet Intervention period: 26 weeks Control group: PA prescription and recommendation: without app to self-monitoring or push notifications Intervention group: push notifications with exercise recommendations and diet tips; app with specific functionalities: self-monitoring of weight at home, gamification, or prescription of PA.
Cristina Lugones-Sanchez (2020) [25]	Spain	Adult women with obesity or classed as overweight	440	IG: 47.4 CG: 48.8	IG: 32.7 ± 3.3 CG: 32.9 ± 3.4	IG: 161 female (69.7%); 70 male (30.3%) CG: 144 female (65.7%); 65 male (34.3%)	Approach of intervention: PA + diet Intervention period: 12 weeks Control group: counselling (5 min) on diet and PA; without app or smart band; single session only. Intervention group: counselling (5 min) on diet and PA; record daily physical activity and food intake daily using app and smart band
Jae-Ho Choi (2023) [26]	Republic of Korea	Adult women	30	IG: 39.7 CG: 39.2	IG: 25.5 ± 4.3 CG: 26.0 ± 4.6	IG: 15 female (100.0%); CG: 15 female (100.0%)	Approach of intervention: PA Intervention period: 12 weeks Control group: None Intervention group: exercise interventions using the mHealth system; app and smart band to track physical activity data
Iris den Uijl (2023) [27]	The Netherlands	Adults with obesity and coronary artery disease or nonvalvular atrial fibrillation	201	IG: 59.0 CG: 59.2	IG: 34.4 ± 4.7 CG: 34.1 ± 4.6	IG: 52 female (33.3%); 68 male (66.7%) CG: 21 female (21.2%); 78 male (78.8%)	Approach of intervention: PA + diet Intervention period: 48 weeks Control group: aerobic training with mainly weight-bearing exercises; without activity tracker, weekly sessions by a dietitian Intervention group: aerobic training with mainly non-weight-bearing exercises and nutrition education by dietician; app and activity monitor to track physical activity data
Daniel Berglind (2020) [28]	Sweden	Adults with mobility disability	110	IG: 35.6 CG: 34.5	IG: 26.3 ± 5.7 CG: 27.2 ± 5.2	IG: 47 female (85.0%); 8 male (15.0%) CG: 43 female (78.0%) 12 male (12.0%)	Approach of intervention: PA + diet Intervention period: 12 weeks Control group: 12-week supervised aerobic/strength training; lifestyle coaching (three sessions); without apps or wearable devices Intervention group: three consultation sessions; using apps to track steps and home-based bodyweight exercise; using food photography app to monitor diet
Jomme Claes (2020) [29]	Belgium and Ireland	Adults with CVD	120	61.4	27.9 ± 4.5	IG: 11 female (18.3%); 49 male (81.7%) CG: 11 female (18.3%); 49 male (81.7%)	Approach of intervention: PA Intervention period: 24 weeks Control group: verbal lifestyle advice; without app or remote support Intervention group: PATHway system, including PA planning, PA intervention, and monitor activity data
Maureen C. Ashe (2015) [30]	Canada	Inactive adult women	20	IG: 63.1 CG: 64.8	IG: 32.9 ± 6.8 CG: 26.9 ± 6.8	IG: 8 female (100.0%) CG: 12 female (100.0%)	Approach of intervention: PA Intervention period: 24 weeks Control group: monthly non-exercise education sessions; without PA prescription or Fitbit; without exercise professional contact Intervention group: activity monitor to record daily step counts, distance walked, and provides immediate feedback on activities; individualized physical activity prescription; education and incentives
Elizabeth J. Lyons (2016) [31]	USA	Adults with obesity or classed as overweight	40	61.48	30.3 ± 3.5	IG: 17 female (85.0%) CG: 17 female (85.0%)	Approach of intervention: PA Intervention period: 12 weeks Control group: None Intervention group: activity monitor and app to set step goals and monitor activity data; consultation; social interaction
Bonnie Spring (2024) [32]	USA	Adults with obesity or classed as overweight	342	IG: 40.9 CG: 40.2	IG: 34.5 ± 4.4 CG: 34.3 ± 4.3	IG: 153 female (76.1%); 48 male (23.9%) CG:152 female (76.4%); 47 male (23.6%)	Approach of intervention: PA + diet Intervention period: 48 weeks Control group: WFS: app and activity monitor with automated feedback to monitor activity data and self-reported diet; without coaching; re-randomization for nonresponses Intervention group: WFS: app and activity monitor with automated feedback to monitor activity data and self-reported diet; with coaching

Abbreviations: PA, physical activity; BMI, body mass index; IG, intervention group; CG, control group;CVD, cardiovascular disease; WFS, a wireless feedback system; PATHway system, a multi-component technology platform that integrates the core components of cardiac rehabilitation and incorporates the four key focus areas of telerehabilitation: telemonitoring, e-learning, telecoaching, and social networking. Notes: age is expressed as mean (years).; BMI is expressed as mean ± SD; *n* = number of participants.

## Data Availability

All data generated or analyzed during this study are included in this published article.

## Abbreviations

The following abbreviations are used in this manuscript:

PA	Physical Activity
IG	Intervention Group
CG	Control Group
RCT	Random Control Trails
ES	Effect Size
BW	Body Weight
BMI	Body Mass Index
BFP	Body Fat Percentage
WHR	Waist-to-hip Ratio
BFM	Body Fat Mass
NR	No Report
NA	No Application

## Appendix A

**Table A1:** Characteristics of physical activity and dietary intervention and key findings of the 9 included studies.

Study	PA and Dietary Intervention		Effect of Intervention on Weight Status					Key Findings
	PA	Diet	BW	BMI	BFP	WHR	BFM	
Alberto Hernández-Reyes (2020) [24]	Push notifications: exercise recommendations; app: specific functionalities: self-monitoring of weight at home, gamification, or prescription of PA	Push notifications: health tips, such as nutritional properties of specific foods	→	→	↓	NR	NR	The intervention group achieved significantly greater fat mass loss compared to the control group, though weight and BMI reductions were similar between groups.
Cristina Lugones-Sanchez (2020) [25]	Counselling: gave advice on physical activity App and smart band: record daily physical activity	Counselling: gave advice on healthy diet App: food intake daily	↓	↓	→	→	→	The mHealth intervention combining a smartphone app and smart band demonstrated greater reductions in weight, BMI, body fat percentage, and fat mass compared to standard counseling alone. No significant changes were observed in waist-to-hip ratio.
Jae-Ho Choi (2023) [26]	App and smart band: track physical activity data; mHealth system: exercise interventions	NA	→	→	↓	→	↓	The 12-week mHealth exercise intervention significantly reduced body fat percentage and fat mass in obese women but did not significantly affect body weight, BMI, or waist-to-hip ratio.
Iris den Uijl (2023) [27]	Group intervention: aerobic training with mainly non-weight-bearing exercises App and activity monitor: track physical activity data	Group intervention: nutrition education by dietician	→	NR	NR	NR	NR	The intervention demonstrated short-term (3 month) improvements in weight loss and physical activity compared to standard CR, but these benefits were not sustained long-term.
Daniel Berglind (2020) [28]	App: step tracking, home-based bodyweight exercise	App: food photograph	→	↓	NR	NR	→	Both the app-based and supervised exercise interventions showed comparable improvements in waist circumference and fat mass, with no significant between-group differences in weight or BMI after 12 weeks.
Jomme Claes (2020) [29]	PATHway system: encourage to achieve the PA goal, activity, and monitor activity data	NA	NR	→	→	→	NR	The intervention helped maintain stable cardiovascular risk factors, including body weight, BMI, body fat percentage, and waist-hip ratio, while these measures showed unfavorable trends in the usual care group over six months. No significant between-group differences were observed in absolute changes, though the intervention group demonstrated better stability in metabolic health markers.
Maureen C. Ashe (2015) [30]	Individualized physical activity prescription; activity monitor: provides immediate feedback on activities and monitor activity data	NA	↓	NR	NR	NR	NR	The intervention group showed significant improvements in weight and diastolic blood pressure compared to the control group, suggesting that reducing sedentary behavior and increasing daily activity may positively influence body composition and cardiovascular health.
Elizabeth J Lyons (2016) [31]	Activity monitor and app: set step goals, idle alert, and monitor activity data	NA	→	NR	→	NR	NR	The intervention showed small but favorable effects on weight and body composition (BMI, body fat), though changes were not statistically significant.
Bonnie Spring (2024) [32]	App and activity monitor: monitor activity data and automated feedback	App: self-reported diet	↓	NR	NR	NR	NR	Participants using the wireless feedback system (WFS) with coaching achieved greater weight loss and BMI reduction compared to WFS alone, though no significant differences were observed in step-up interventions for non-responders.

Abbreviations: PA, physical activity; BW, body weight; BMI, body mass index; WHR, waist-to-hip ratio; BFP, body fat percentage; BFM, body fat mass; NR, not reported; NA, not application; PATHway system, a multi-component technology platform that integrates the core components of cardiac rehabilitation and incorporates the four key focus areas of telerehabilitation: telemonitoring, e-learning, telecoaching, and social networking. Note: ↓, or → = If the study had a control group, significant worsening or no effect on outcome was compared the intervention group with the control group; if the study did not have a control group, significant improvement, worsening, or no effect on outcome at pre and post the intervention.

**Table A2:** Meta-analysis of GPS-enabled PA interventions on weight reduction by the characteristic of interventions.

	Sample Size	Number of Studies	Meta-Analytic Effect Size			Heterogeneity		
			Effect Size (95% CI)	Z-Value	*p*-Value	*I*^2^ (%)	Q	*p*-Value ^a^
A. BW								
**Total**	1243	8	−0.241 (−0.356, −0.127)	−4.133	<0.001	6.5%	7.49	0.380
**Intervention type**								
PA	90	3	−0.328 (−0.616, −0.039)	−2.228	0.026	0.0%	0.21	0.902
PA + diet	1153	5	−0.208 (−0.372, −0.044)	−2.481	0.013	42.1%	6.91	0.141
**Gender**								
Male and female	1133	5	−0.224 (−0.381, −0.067)	−2.791	0.005	44.0%	7.14	0.128
Only female	110	3	−0.275 (−0.653, 0.102)	−1.429	0.153	0.0%	0.32	0.853
**Age ***								
≤60	1183	6	−0.221 (−0.372, −0.070)	−2.877	0.004	31.2%	7.26	0.202
>60	60	2	−0.304 (−0.618, 0.010)	−1.899	0.058	0.0%	0.07	0.791
**Intervention period**								
≤3 month	623	4	−0.239 (−0.386,−0.092)-	−3.182	0.001	0.0%	2.10	0.552
≥6 month	620	4	0.221 (−0.469, 0.026)	−1.751	0.080	44.2%	5.38	0.146
**B. BMI**								
**Total**	760	5	−0.185 (−0.375, 0.005)	−1.911	0.056	26.7%	5.46	0.243
**Intervention type**								
PA	150	2	−0.182 (−0.504, 0.141)	−1.105	0.269	0.0%	0.01	0.919
PA + diet	610	3	−0.152 (−0.477, 0.172)	−0.920	0.358	62.8%	5.38	0.068
**Gender**								
Male and female	670	3	−0.153 (−0.440, 0.134)	−1.044	0.296	63.1%	5.42	0.067
Only female	90	2	−0.199 (−0.614, 0.215)	−0.941	0.347	0.0%	0.03	0.866
**Age ***								
≤60	640	4	−0.161 (−0.424, 0.102)	−1.202	0.229	44.7%	5.42	0.143
>60	120	1	−0.190 (−0.551, 0.171)	−1.033	0.302	-	-	-
**Intervention period**								
≤3 month	580	3	0.163 (−0.211, 0.538)	−0.699	0.485	63.1%	5.42	0.066
≥6 month	180	2	−0.202 (−0.496, 0.093)	−1.343	0.179	0.0%	0.01	0.914
**C. BFP**								
**Total**	690	5	−0.412 (−0.804, −0.020)	−2.059	0.039	76.0%	16.66	0.002
**By type of intervention**								
PA	190	3	−0.425 (−1.091, 0.240)	−1.253	0.210	75.8%	8.27	0.016
PA + diet	500	2	−0.477 (−1.292, 0.338)	−1.147	0.251	88.0%	8.34	0.004
**Gender**								
Male and female	600	3	−0.090 (−0.250, 0.070)	−1.103	0.270	0.0%	0.18	0.912
Only female	90	2	−1.051 (−1.495, −0.606)	−4.631	<0.001	0.0%	0.59	0.441
**Age ***								
≤60	530	3	−0.716 (−1.492, 0.061)	−1.807	0.071	87.1%	15.56	<0.001
>60	160	2	−0.067 (−0.374, 0.240)	−0.430	0.667	0.0%	0.16	0.692
**Intervention period**								
≤3 month	510	3	−0.427 (−1.032, 0.178)	−1.385	0.166	76.2%	8.39	0.015
≥6 month	180	2	−0.459 (−1.344, 0.425)	−1.017	0.309	86.8%	7.56	0.006

PA, physical activity; BW, body weight; BMI, body mass index; BFP, body fat percentage; *I*^2^ > 50% or *p* value < 0.10 was considered evidence of heterogeneity; *p* value ^a^ referred to the results of Cochran’s Q test for heterogeneity within each subgroup. * The determination was based on the median or mean of the baseline age.

**Table A3:** Sensitivity analysis of weight, BMI, and body fat percentage.

Study	ES	[95% Conf. Interval]	*I*^2^ (%)	*p*
A. BW				
Alberto Hernández-Reyes (2020) [24]	−0.238	−0.369, −0.106	19.8	0.279
Cristina Lugones-Sanchez (2020) [25]	−0.226	−0.382, −0.069	19.6	0.280
Jae-Ho Choi (2023) [26]	−0.232	−0.357, −0.107	16.3	0.306
Iris den Uijl (2023) [27]	−0.288	−0.405, −0.171	0.0	0.684
Daniel Berglind (2020) [28]	−0.266	−0.379, −0.154	0.0	0.449
Maureen C. Ashe (2015) [30]	−0.238	−0.397, −0.180	19.7	0.279
Elizabeth J Lyons (2016) [31]	−0.225	−0.360, −0.091	17.5	0.296
Bonnie Spring (2024) [32]	−0.188	−0.312, −0.063	0.0	0.631
**B. BMI**				
Alberto Hernández-Reyes (2020) [24]	−0.162	−0.401, 0.076	45.1	0.141
Cristina Lugones-Sanchez (2020) [25]	−0.070	−0.291, 0.150	0.0	0.509
Jae-Ho Choi (2023) [26]	−0.175	−0.399, 0.049	44.6	0.144
Daniel Berglind (2020) [28]	−0.287	−0.443, −0.133	0.0	0.872
Jomme Claes(2020) [29]	−0.161	−0.424, 0.102	44.7	0.143
**C. BFP**				
Alberto Hernández-Reyes (2020) [24]	−0.257	−0.615, 0.101	66.0	0.032
Cristina Lugones-Sanchez (2020) [25]	−0.558	−1.136,0.021	77.7	0.004
Jae-Ho Choi (2023) [26]	−0.256	−0.558, 0.076	66.5	0.030
Jomme Claes(2020) [29]	−0.562	−1.116, −0.007	80.8	0.001
Elizabeth J Lyons (2016) [31]	−0.483	−0.960, −0.006	82.0	<0.001

ES, effect size; BW, body weight; BMI, body mass index; BFP, body fat percentage; *I*^2^ > 50% or *p* < 0.10 was considered evidence of heterogeneity.

**Table A4:** Assessment of publication bias by Egger’s test and Begg’s test.

	BW	BMI	BFP
*p* (Egger’s test)	0.698	0.201	0.154
*p* (Begg’s test)	0.902	0.462	0.221

BW, body weight; BMI, body mass index; BFP, body fat percentage.
